# Quantitative evaluation of the biomechanical and viscoelastic properties of the dog patellar tendon in response to neuromuscular blockade at different stifle angles

**DOI:** 10.1371/journal.pone.0292453

**Published:** 2024-01-02

**Authors:** Dito Anggoro, Melpa Susanti Purba, Norihiro Nishida, Harumichi Itoh, Kazuhito Itamoto, Yuki Nemoto, Munekazu Nakaichi, Hiroshi Sunahara, Kenji Tani

**Affiliations:** 1 Laboratory of Veterinary Surgery, Joint Graduate School of Veterinary Medicine, Yamaguchi University, Yamaguchi, Japan; 2 Department of Surgery and Radiology, Faculty of Veterinary Medicine, Gadjah Mada University, Yogyakarta, Indonesia; 3 Department of Orthopedic Surgery, Graduate School of Medicine, Yamaguchi University, Yamaguchi, Japan; 4 Laboratory of Small Animal Clinical Science, Joint Faculty of Veterinary Medicine, Yamaguchi University, Yamaguchi, Japan; 5 Laboratory of Veterinary Radiology, Joint Faculty of Veterinary Medicine, Yamaguchi University, Yamaguchi, Japan; 6 Laboratory of Veterinary Surgery, Joint Faculty of Veterinary Medicine, Yamaguchi University, Yamaguchi, Japan; Yarmouk University, JORDAN

## Abstract

The patellar tendon (PT) is crucial for maintaining stability and facilitating movement in the stifle joint. Elastography has been recognized as a prominent method for evaluating PT properties in humans and dogs. The utilization of oscillation methods in canine studies remains limited despite their extensive documentation in human studies. Our study represents the first effort to quantitatively assess and compare the effects of muscle relaxant on the biomechanical and viscoelastic characteristics of the PT at varying stifle angles in living dogs. Five healthy female beagles were used in this study. Biomechanical (tone, stiffness, and decrement) and viscoelastic (relaxation time and creep) properties of the PT were measured using MyotonPRO (Myoton Ltd, Estonia) prior to and following administration of rocuronium (0.5 mg/kg/body weight) at normal, extended, and flexed positions. Rocuronium was selected for its safety, controllability, and widespread clinical use in veterinary anesthesia. Two-way analysis of variance showed that tone, stiffness, and decrement were significantly higher (P < 0.001) in the control group than in the muscle relaxation group. At the same time, relaxation time and creep were significantly lower (P < 0.001) in the control group than in the muscle relaxation group. The findings indicate that stifle angle position and muscle rexalant administration fundamentally alter the biomechanical loading conditions of the PT, leading to changes in its viscoelastic properties. Therefore, this novel quantitative data could benefit clinical settings that necessitate accurate and objective methods for risk identification and monitoring PT biomechanics in dogs.

## Introduction

Tendons are mechanical cushions that transmit and absorb force when jumping and landing. Tendons also contribute to storing and releasing energy, which helps muscles function more effectively [1]. Knee extensors comprise the quadriceps muscle and tendon, medial and lateral patellar retinaculum, patella, patellar tendon (PT), and tibial tubercle [2]. The PT also plays a crucial role in transferring the force from the quadriceps to the tibia [3]. The proximal attachment (inferior pole) is mainly affected if the PT becomes inflamed from constant strain. Meanwhile, the superior pole of the patella (quadriceps tendon insertion) and tibial tuberosity (patellar tendon distal attachment) are rarely affected by strain [4]. Hence, accurately assessing tendon properties may be a valuable addition to the standard tests to evaluate skeletal muscle function.

Numerous methods for diagnosing canine PT disorders, including radiography, magnetic resonance imaging (MRI), and elastography, are used in clinical practice. Radiography may reveal soft tissue edema, irregular margins, and the PT’s thickening at the tibial tuberosity level. Meanwhile, MRI and elastography provide better accuracy and image quality when diagnosing injuries due to their superior visualization of soft tissues [5]. However, the availability and dependability of these modalities depend on the laboratory settings, non-quantitative analysis methods, and operator skills or experience. Alternatively, myotonometric medicine has been widely used in human evaluation to evaluate the PT, Achilles tendon (AT), and viscoelastic muscles [1, 6–9]. Myotonometric evaluations are based on the oscillation theory that involves the application of several mechanical impulses to soft tissue and measuring the reflected energy, representing the tissue’s viscoelastic properties. The device has several advantages: it is portable, non-invasive, painless, and highly reliable [10, 11].

Neuromuscular blocking agents (NMBA) are frequently used to improve skeletal muscle relaxation during surgery that requires general anesthesia. Rocuronium is an example of a non-depolarizing NMBA frequently used in veterinary clinical practice because of its ability to induce muscle paralysis while reducing stiffness. Rocuronium relaxes the muscles by acting on nerve endings and inhibiting the release of acetylcholine. Additionally, rocuronium improves tendon gliding and joint movement, resulting in a safer and more efficient surgery [12]. The angle of the stifle joint would significantly impact the PT and its viscoelastic properties. Studies regarding PT biomechanics and viscoelastic properties have shown that changes in the stifle flexion angle can significantly impact the PT’s tone, stiffness, and elasticity. In humans, it is hypothesized that knee joint position plays a role in determining the elasticity of the PT, representing the highest elasticity at flexion using the elastography method [13]. Reportedly, flexion angles of 90° resulted in the highest PT tone and stiffness in both dominant and non-dominant legs [8]. Other studies reported that elastography assessment of the patellar ligament demonstrated a considerably lower percentage of stiffness in the standing position (135°) than in other positions [14]. Even though human and canine knees are not anatomically identical, a prior investigation discovered sufficient biomechanical similarities to justify comparing specific characteristics [15, 16].

To the best of our knowledge, in live animals (not cadavers), no studies have addressed all five parameters included in the Myoton device on dog PT. These parameters include muscular tone, dynamic stiffness, logarithmic decrement, relaxation time, and creep. Therefore, this study aimed to investigate PT biomechanical and viscoelastic properties in response to the effects of muscle relaxants and under different knee loading angles (normal position, flexion, and extension). Analysis of a dog’s PT using the Myoton device could provide reference values on PT biomechanical and viscoelastic properties in various circumstances. These findings may offer an objective diagnostic procedure or comparison between the various examination techniques. We hypothesized that the biomechanical and viscoelastic properties of the patellar tendon (PT) in dogs will exhibit significant variations under the influence of the muscle relaxant, particularly in response to changes in stifle joint angles.

## Materials and methods

### Animals

Five adult female Beagles (B1, B2, B3, B4, and B5) with a mean age of 10.6 ± 0.55 years (range, 10–11 years), mean body weight of 11.4 ± 0.74 kg (range, 10.5–12.2 kg), and body condition score of 3 were used in this study. The dogs were provided by the Yamaguchi University Animal Medical Centre as experimental dogs and had no 1) history of lameness, 2) relationship to any orthopedic examination, and 3) radiographic evidence of joint disease. The protocol was approved by the Committee on the Ethics of Animal Experiments of the Yamaguchi University, Japan (Protocol Number: 539).

### Anesthesia and monitoring

Anesthesia was administered to all dogs as it would be easier to manipulate the various stifle angle positions during the examination to create a condition almost identical to those obtained from living dogs. All dogs were fasted for at least 12 hours before receiving anesthesia. The dog’s left and right cephalic veins were catheterized using a 22-gauge catheter (Terumo Co., Ltd., Tokyo, Japan) for intravenous infusion of fluids and rocuronium. Ringer’s acetate solution (5 mL/kg/hour, Veen F, Fuso Pharmaceutical Industries, Ltd., Japan) was administered with a precision infusion pump (TOP-3300; TOP Corporation).

Induction was started with propofol 1% (Mylan®, Pfizer, Japan) at 7 mg/kg intravenously. An 8 mm endotracheal tube was inserted into the trachea and connected to an anesthetic machine (Dräger Fabius® Plus, Germany) and maintained with isoflurane (Mylan®, Pfizer, Japan) delivered via a 2-liter rebreathing bag. The anesthetic depth was controlled by maintaining isoflurane within 1.5–2.0 minimum alveolar concentration. To ensure the dogs were in stage III plane 3, jaw tone, eye position, pupil size, and several reflexes, including palpebral, swallowing, and pedal reflexes, were assessed. During the experiment, several cardiopulmonary variables that were measured by electrocardiography, pulse oximetry, capnography, temperature, and oscillometric non-invasive blood pressure were monitored using a multiparameter monitor (BSM-6301, Life Scope, Nihon Kohden, Japan) and noted on the anesthetic record every 5 minutes by the anesthetist. The body temperature was maintained between 37.5°C and 38.0°C with a patient warming system (775, 3M™ Bair Hugger™).

### Radiographic evaluation

Standard mediolateral radiographs of the stifle joint in the normal, extended, and flexed positions were taken using RADspeed Pro (Shimadzu, Japan). Due to concerns discussed earlier, the standing position will hereafter be described as a normal position when taking radiographic examinations. The angle of the normal (140°) stifle joint position was estimated based on the leg position of a standing dog. Maximal flexion (34°) and extension (135°) were performed on both legs. The angle measurements were determined using a universal goniometer (Takumed, Sankei Kanko Co., Ltd.).

**Fig 1:**
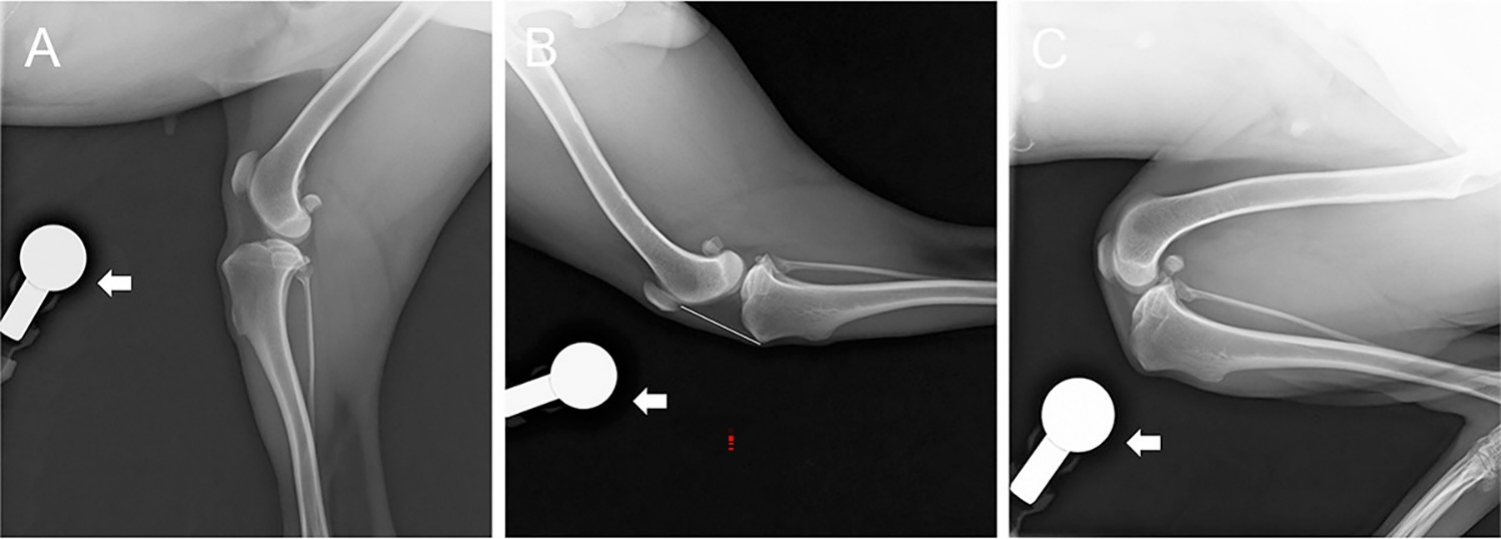
Radiographic comparison of the patellar tendon. (A) normal, (B) extended, and (C) flexed positions. Patellar tendon length (PTL) and radiographic reference ball are shown as white lines and arrows, respectively.

**Fig 2:**
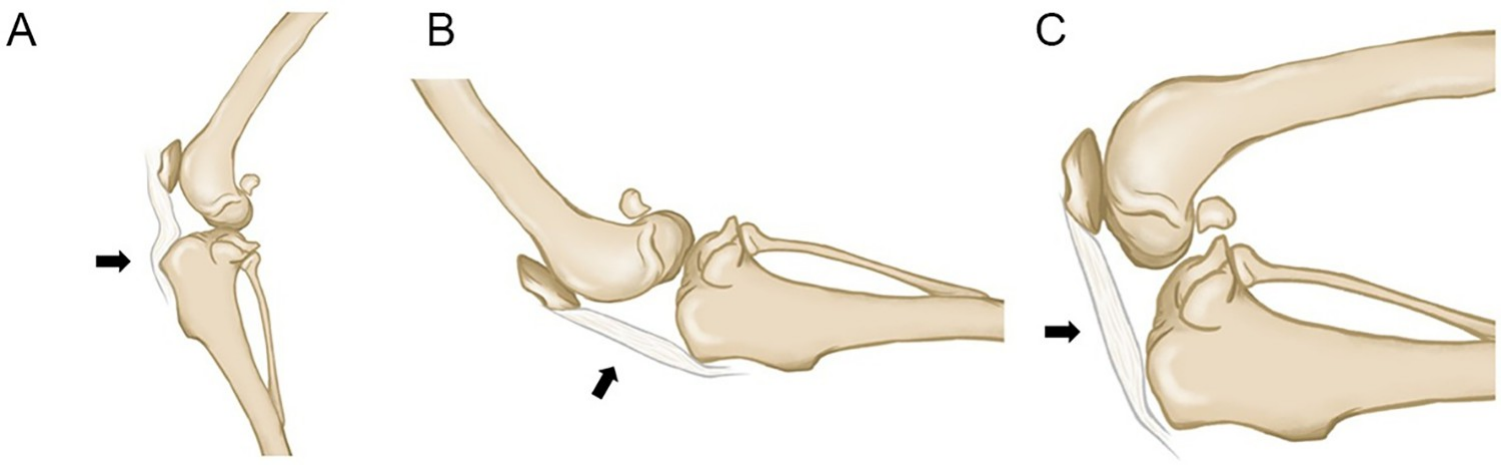
Illustration of the patellar tendon shape (arrow). (A) Normal, (B) extension, and (C) flexion of the stifle joint.

The main criteria for determining acceptable image results were superimposition of the femoral condyles and fabellae of the gastrocnemius muscle and that the fibular head should be positioned caudally along the proximal section of the tibial condyle. The total PT length (PTL) was determined as specified by a previous study [17] and was measured from the distal aspect of the patella to the proximal aspect of the tibial tuberosity (Fig 1). Additionally, an illustration was provided to highlight the changes in PT shape that occur at different stifle angles (Fig 2). The results of PTL on varied stifle angles are demonstrated in Table 1. A radiographic reference ball measuring 25 mm was utilized to reduce scaling error during measurement. This examination was performed using SYNAPSE QA (Fujifilm, Japan).

**Table 1:** Patellar tendon length (PTL) during various stifle joint conditions.

Position	Patellar length (mm)				
	B1	B2	B3	B4	B5
Normal	27.1	28.0	28.1	23.8	24.5
Extension	30.0	30.6	32.8	27.8	27.4
Flexion	28.8	29.5	31.7	26.9	26.4

Abbreviations: mm (millimeters).

### Neuromuscular blockade and monitoring

Before administering muscle relaxant, neuromuscular function was evaluated on a thoracic limb through responses to train-of-four (TOF) stimulation utilizing a muscle relaxation module (AF-201P, Nihon Kohden, Japan) in conjunction with a display monitor (VA-201R, Nihon Kohden, Japan). The dog was positioned in lateral recumbency, and the extended limb was positioned parallel to the table with the antebrachium extended. At the level of the medial epicondyle of the humerus, two needles were implanted subcutaneously across the ulnar nerve and positioned 2 cm apart. Meanwhile, alligator clips (101P-B, K942B, Nihon Kohden, Japan) secured both needles to the TOF muscle relaxation module. The acceleration transducer (TA-101P, P036A, Nihon Kohden, Japan) was applied to the palmar aspect of the paw using surgical tape (Micropore Surgical Tape, 2.54 cm wide; 3M Health Care), and carpus flexion was elicited. A temperature sensor (TT-101P, P036B, Nihon Kohden, Japan) was inserted into the gingiva of the upper lips.

Stabilization and calibration were performed before the administration of rocuronium (Eslax®, MSD Connect, Japan). Signal stabilization was performed using a TOF sequence every 15 seconds (2 Hz, 50 mA) for 10 minutes to facilitate twitch potentiation. Moreover, the monitor’s built-in automatic calibration function calibrated the accelerometric measurement. At the beginning of the assessment and baseline, the response remained stable for 5–10 minutes, and a bolus dose of rocuronium (0.5 mg/kg) was administered intravenously. Immediately after, a maintenance dose was administered at 0.2 mg/kg/hr using a precision syringe infusion pump (TOP-5530; TOP Co., Ltd.). The infusion rate was adjusted in increments ranging from 0.2 to 0.5 mg/kg/hour, with at least 2 minutes between each adjustment. The blockade was successful when none of the four twitches could be observed. Infusion was terminated once all measurements were completed. Subsequently, the TOF ratio was monitored until it returned to >90% to ensure that the effects of muscle relaxant had worn off before discontinuing isoflurane administration.

### Procedure of assessment

A hand-held MyotonPRO device (MyotonPRO, Myoton Ltd, Estonia) was programmed to perform a series of scans at regular intervals every second, each of which would consist of a short-duration (15 ms) impulse using a minimum amount of mechanical force (0.4 N) and gentle precompression (0.18 N). Based on the mechanical dynamic response method, it will provide a subsequent computation of parameters characterizing the PT’s biomechanical (tissue/muscle tone, dynamic stiffness, and decrement) and viscoelastic properties (mechanical stress relaxation time and ratio of relaxation and deformation time).

The multi-scan measurement consists of five individual measurements (the instrument generated five rapid mechanical impulses) and is automatically reported as the average of these observations. Multi-scan measurements were conducted five times for the three different stifle angles on both legs and groups. Following this, analysis was performed using the average of the five repetitions. All dogs were positioned in lateral recumbency, and all measurements were performed by a single examiner (DA). Moreover, to prevent misinterpretation due to the stability factor, the leg that rested on the examination table was utilized as the standard for measuring.

The same dog was subjected to two experimental procedures. All dogs were evaluated prior to rocuronium administration; this group is the control group. After completing all evaluations, rocuronium was delivered to the same dog, after which they represented the muscle relaxation group and underwent evaluations similar to those previously mentioned. The overlying hair on both patellar ligaments was clipped to avoid any potential bias. The PT was determined by determining the midpoint of the distance between the distal pole of the patella and the tibial tuberosity according to each stifle joint position angle. A standard probe (3 mm) was directed to the designated area and measured the PT by lightly applying the contact detection sensor perpendicularly to the measurement surface. The measurements were repeated when the coefficient of variation was >3%. Nevertheless, the results were retained in every other circumstance. The mean data were used for statistical analysis.

### Parameters

The Myoton device provides five distinct indicators to ensure accurate and reliable outcomes, including tone/oscillation/frequency (Hz, Hertz), stiffness (N/m, Newton/meter), decrement/elasticity (log, logarithmic decrement), relaxation time (ms), and creep (Deborah number [dn]). Tone signifies the tension when a tissue remains passive at rest or without voluntary contraction. The stiffness value is computed as the maximal oscillation acceleration and tissue deformation sensed by the transducer. The ability of soft tissues to return to their original shape after being deformed is referred to as elasticity. Furthermore, elasticity can be considered the inverse of tissue oscillational dampening (the larger the decrement, the lower the elasticity). Relaxation time is defined as the time required for the tissue to recover after being displaced. Creep denotes the constant lengthening of tissue in response to repeated tensile stress (ratio of relaxation and deformation time).

### Statistical analysis

Mean values and standard deviation (SD) were used to explain the information acquired from the assessment. Two-way analysis of variance (ANOVA) with repeated measures ANOVA (RM ANOVA) was used to analyze the effect of treatments (control and muscle relaxation) and stifle angles (normal position, extension, and flexion) on the changes in the biomechanical (tone, stiffness, and decrement) and viscoelastic (relaxation time and creep) characteristics of the PT. The Shapiro–Wilk test was used to determine the normal data distribution. The interaction effect between different stifle angles and the effects of muscle relaxants on the biomechanical and viscoelastic characteristics of the PT was analyzed with the same method. Bonferroni test was performed for post hoc comparisons. Mauchly’s test of sphericity was used to evaluate the variances of the differences between all combinations of the related groups (analysis of sphericity). The Greenhouse–Geisser assumption was performed to analyze the interaction when sphericity was violated. Pearson correlation analysis was used to observe the relationship between dog body weight and PT length. SPSS version 27 (IBM SPSS Statistics, version 27, SPSS Inc, Chicago, IL, USA) was used for all analyses. Differences were significant at P < 0.05.

## Results

Table 2 shows the descriptive statistics for PT parameters contributing to its biomechanical and viscoelastic properties.

**Table 2:** Descriptive statistics for the biomechanical and viscoelastic characteristics of the patellar tendon.

Variable		Control			Muscle relaxation		
		Normal	Extension	Flexion	Normal	Extension	Flexion
Tone (Hz)	Mean	22.99^e^	27.66^a^^,^^e^	31.61^a^^,^^b^^,^^e^	18.81	24.67^c^	27.68^c^^,^^d^
	SD	0.99	0.55	0.46	0.44	0.54	0.53
	Range	3.70	2.07	1.71	1.70	2.08	2.02
	95% CI	22.58–23.40	27.43–27.89	31.42–31.80	18.62–18.99	24.44–24.89	27.45–27.90
Stiffness (N/m)	Mean	481^e^	745^a^^,^^e^	943^a^^,^^b^^,^^e^	456	635^c^	765^c^^,^^d^
	SD	4.28	1.64	1.40	2.01	1.79	1.34
	Range	16.37	6.22	5.31	7.15	6.30	5.79
	95% CI	480–483	745–746	942–944	455–457	634–636	765–766
Decrement (log)	Mean	0.63^e^	0.78^a^^,^^e^	1.46^a^^,^^b^^,^^e^	0.49	0.63^c^	1.28^c^^,^^d^
	SD	0.04	0.07	0.03	0.04	0.04	0.02
	Range	0.18	0.26	0.13	0.17	0.16	0.09
	95% CI	0.61–0.65	0.75–0.82	1.44–1.47	0.47–0.51	0.61–0.65	1.27–1.29
Relaxation time (ms)	Mean	10.05^e^	6.38^a^^,^^e^	5.65^a^^,^^b^^,^^e^	12.10	8.36^c^	7.30^c^^,^^d^
	SD	0.38	0.16	0.10	0.71	0.62	0.49
	Range	1.35	0.61	0.39	2.69	2.36	1.80
	95% CI	9.89–10.2	6.31–6.45	5.61–5.70	11.80–12.39	8.10–8.61	7.10–7.51
Creep (dn)	Mean	0.65^e^	0.45^a^^,^^e^	0.40^a^^,^^b^^,^^e^	0.75	0.51^c^	0.49^c^^,^^d^
	SD	0.01	0.01	0.01	0.75	0.51	0.49
	Range	0.06	0.07	0.02	0.07	0.08	0.12
	95% CI	0.64–0.66	0.44–0.46	0.39–0.40	0.74–0.75	0.51–0.52	0.48–0.51

Abbreviations: CI, confidence intervals; SD, standard deviation; Hz: Hertz; N/m: Newton/meter; log: logarithmic; ms: milliseconds; dn: Deborah number.

^a^ Significantly (P < 0.05) different from the value obtained in normal position (control group).

^b^ Significantly (P < 0.05) different from the value obtained in the extension position (control group).

^c^ Significantly (P < 0.05) different from the value obtained in normal position (muscle relaxation group).

^d^ Significantly (P < 0.05) different from the value obtained in the extension position (muscle relaxation group).

^e^ Significantly (P < 0.05) different from the value obtained in other treatments.

### Tone

**Fig 3:**
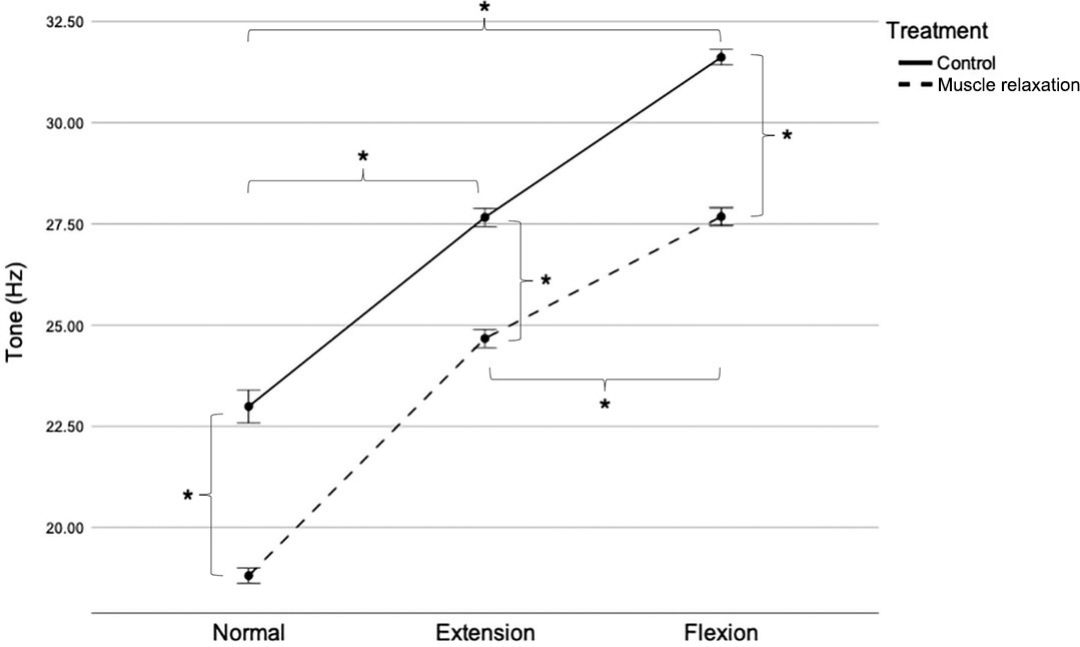
Patellar tendon tone in the control and muscle relaxation groups. Mean with SEM. *P < 0.05. Hz, Hertz; SEM, standard error of the mean.

PT tone values are displayed in Fig 3. Two-way RM ANOVA revealed significant interactions between treatment and position (F [1.4, 34] = 17.987; P < 0.001). The effects of different treatments (control and muscle relaxation) on PT tone depended on stifle angle position. In the normal position, PT tone was significantly higher in the control group (22.99 ± 0.99 Hz) than in the muscle relaxation group (18.81 ± 0.44 Hz) (F [1,24] = 372.825; P < 0.001), with a mean difference of 4.18 Hz (95% CI [3.73, 4.62]). In the extended position, PT tone was significantly higher in the control group (27.66 ± 0.55 Hz) than in the muscle relaxation group (24.67 ± 0.54 Hz) (F [1,24] = 2,361.062; P < 0.001), with a mean difference of 2.99 Hz; (95% CI [2.86, 3.11]). In the flexed position, PT tone was also significantly higher in the control group (31.61 ± 0.46 Hz) than in the muscle relaxation group (27.68 ± 0.53 Hz) (F [1,24] = 1,486.196; P < 0.001), with a mean difference of 3.93 Hz (95% CI [3.72, 4.14]).

The effect of stifle angle position on PT tone value varied depending on the treatment. In the control group, PT tone was lower in the normal position (22.99 ± 0.99 Hz) than in the extended position (27.66 ± 0.55 Hz), with a mean difference of 4.67 Hz (95% CI [4.13, 5.21]). However, PT tone was higher in the flexed position (31.61 ± 0.46 Hz) than in the normal and extended positions, with a mean difference of 8.62 Hz (95% CI [8.04, 9.21]) and 3.95 Hz (95% CI [3.68, 4.22]), respectively (F [1.3,31] = 1,050.686; all P < 0.001). Similarly, in the muscle relaxation group, PT tone was lower in the normal position (18.81 ± 0.44 Hz) than in the extended position (24.67± 0.54 Hz), with a mean difference of 5.86 Hz (95% CI [5.68, 6.03]). PT tone was higher in the flexed position (27.68 ± 0.53 Hz) than in the normal and extended positions, with a mean difference of 8.87 Hz (95% CI [8.68, 9.05]) and 3.01 Hz (95% CI [2.79, 3.22]), respectively (F [2,48] = 7,231.708; all P < 0.001).

### Stiffness

**Fig 4:**
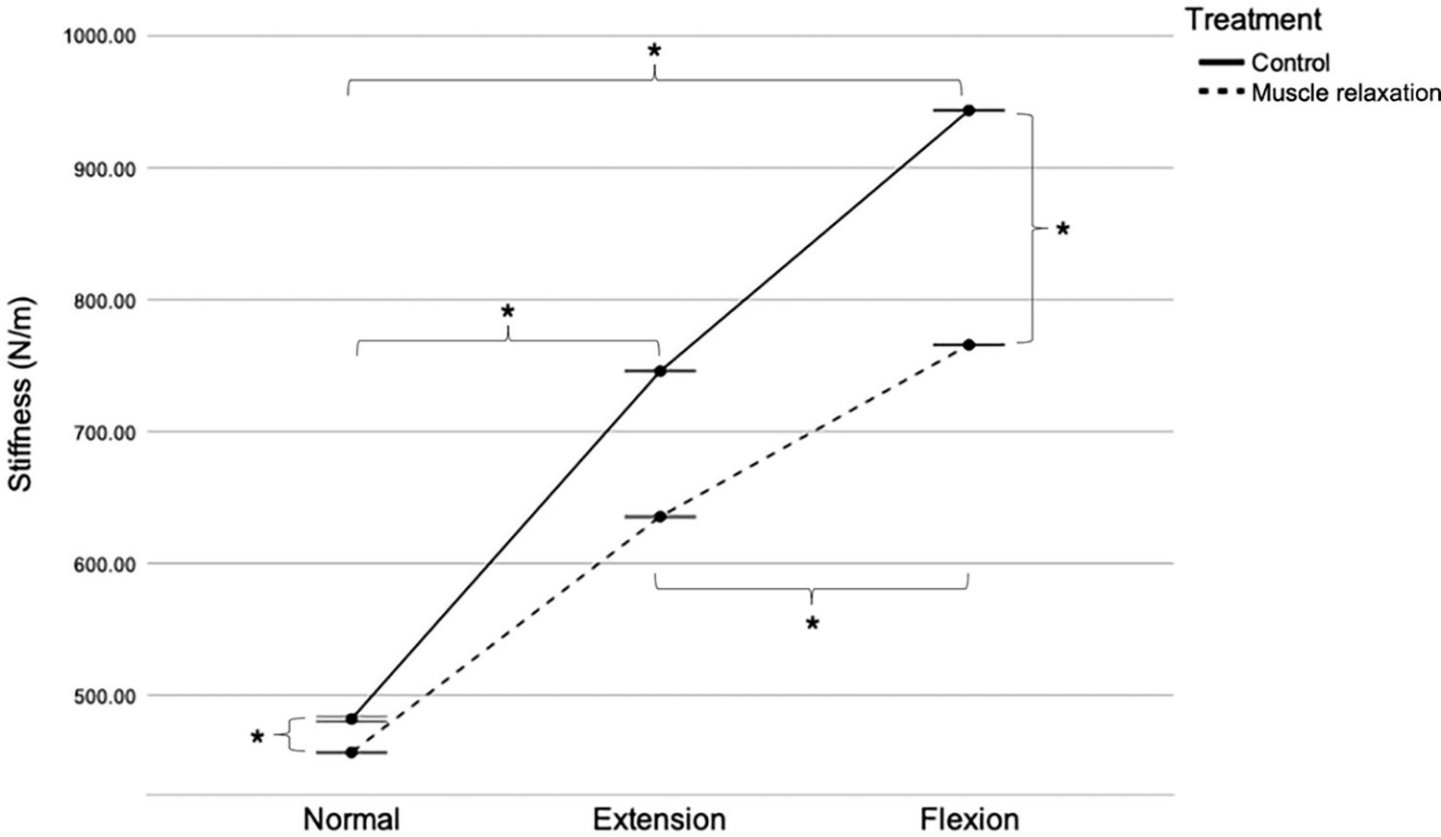
Patellar tendon stiffness in the control and muscle relaxation groups. Mean with SEM. *P < 0.05. N/m, Newton/meter; SEM, standard error of the mean.

The difference in stiffness between the control and muscle relaxation groups in various positions and at different stifle angles is illustrated in Fig 4. Two-way RM ANOVA revealed significant interactions between treatment and position (F [2, 48] = 2,2295.392; P < 0.001). Notably, the effects of treatment on PT stiffness depended on the stifle angle position. In the normal position, PT stiffness was significantly higher in the control group (481 ± 4.28 N/m) than in the muscle relaxation group (456 ± 2.01 N/m) (F [1,24] = 1,206.193; P < 0.001), with a mean difference of 25 N/m (95% CI [23.77, 26.78]). Similarly, in the flexed position, PT stiffness was significantly higher in the control group (745 ± 1.64 N/m) than in the muscle relaxation group (635 ± 1.79 N/m) (F [1,24] = 70,860.065; P < 0.001), with a mean difference of 110 N/m (95% CI [109, 111]). In the flexed position, PT stiffness was also significantly higher in the control group (943 ± 1.40 N/m) than in the muscle relaxation group (765 ± 1.34 N/m) (F [1,24] = 16,1471.953; P < 0.001), with a mean difference of 178 N/m (95% CI [176, 178]).

The interaction also indicated that the effect of stifle angle position on PT stiffness varied depending on the treatment. In the control group, PT stiffness was lower in the normal (481 ± 4.28 N/m) than in the extended (745 ± 1.64 N/m) position, with a mean difference of 264 N/m (95% CI [261, 266]). However, PT stiffness was higher in the flexed position (943 ± 1.40 N/m) than in the normal and extended positions, with a mean difference of 462 N/m (95% CI [459, 463]) and 198 N/m (95% CI [196, 198]), respectively (F [1.2,30] = 152,646.443; all P < 0.001). Similarly, in the muscle relaxation group, PT stiffness was lower in the normal (456 ± 2.01 N/m) than in the extended (635 ± 1.79 N/m) position, with a mean difference of 179 N/m (95% CI [177, 180]). PT stiffness was higher in the flexed positions (765 ± 1.34 Hz) than in the normal and extended positions, with a mean difference of 309 N/m (95% CI [307.774, 310.230]) and 130 N/m (95% CI [129, 131]), respectively (F [2,48] = 18,7771.155; all P < 0.001).

### Decrement

**Fig 5:**
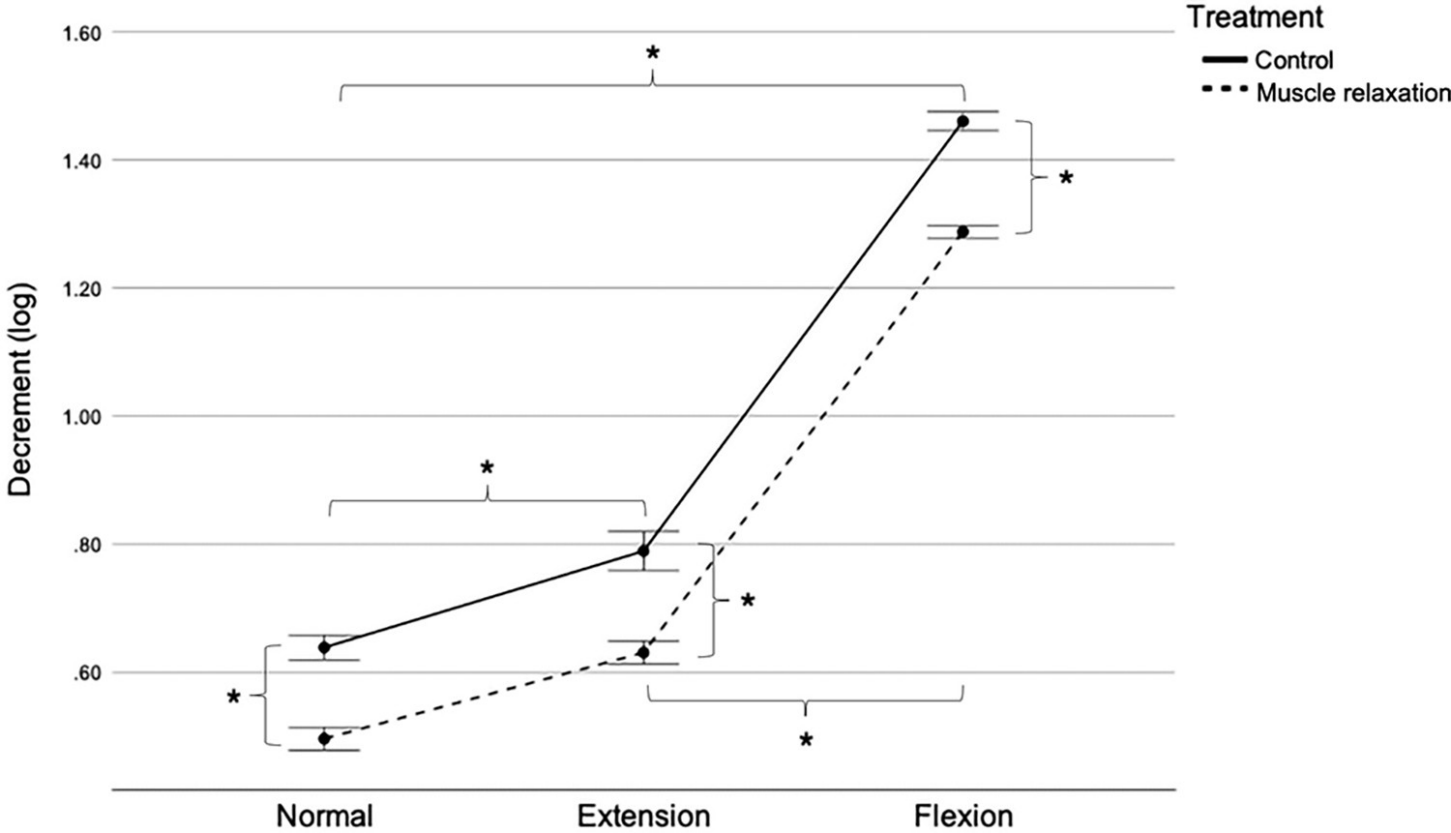
Patellar tendon decrement in the control and muscle relaxation groups. Mean with SEM. *P < 0.05. log, logarithmic; SEM, standard error of the mean.

Two-way RM ANOVA indicated no significant interaction effect (F [2,48] = 2.267; P = 0.115), indicating no combined effect for treatment and position on decrement as depicted in Fig 5. However, both main effects of treatment and position were statistically significant (P < 0.001). The main effect of treatment showed a statistically significant difference in decrement between treatments across all positions (F [1, 24] = 689.039; P < 0.001). Moreover, the main effect of position showed a statistically significant difference in decrement between all positions across all treatments (F [2, 48] = 4,310.934; P < 0.001).

### Relaxation time

**Fig 6:**
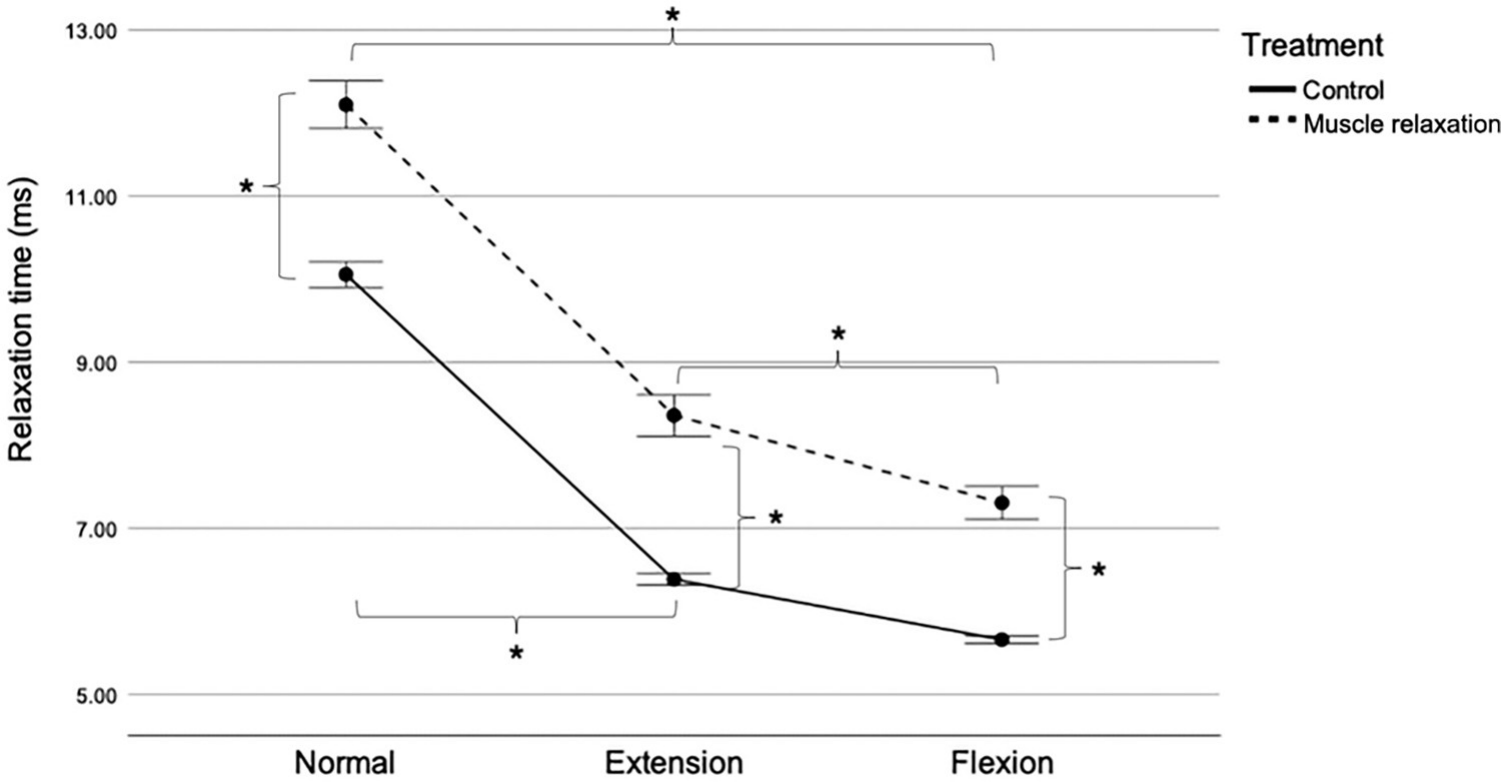
Patellar tendon relaxation time in the control and muscle relaxation groups. Mean with SEM. *P < 0.05. ms, milliseconds; SEM, standard error of the mean.

Fig 6 illustrates the difference in relaxation time between the control and muscle relaxation groups at various stifle angle positions. Two-way RM ANOVA revealed significant interactions between treatment and position (F [1.3, 31] = 3,428; P = 0.04). The effects of the different treatments on the PT relaxation time depended on the stifle angle position. In the normal position, PT relaxation time was significantly shorter in the control group (10.05 ± 0.38 ms) than in the muscle relaxation group (12.10 ± 0.71 ms) (F [1,24] = 189.320; P < 0.001), with a mean difference of 2.05 ms (95% CI [1.73, 2.35]). Similarly, in the extended position, PT relaxation time was significantly shorter in the control group (6.38 ± 0.16 ms) than in the muscle relaxation group (8.36 ± 0.62 ms) (F (1,24) = 184.204; P < 0.001), with a mean difference of 1.98 ms (95% CI [1.67, 2.27]). In the flexed position, PT relaxation time was also significantly shorter in the control group (5.65 ± 0.10 ms) than in the muscle relaxation group (7.30 ± 0.49 ms) (F [1,24] = 241.977; P < 0.001), with a mean difference of 1.65 ms (95% CI [1.43, 1.86]).

The interaction also indicated that the effect of stifle angle position on PT relaxation time varied depending on the treatment. In the control group, PT relaxation time was longer in the normal (10.05 ± 0.38 ms) than in the extended (6.38 ± 0.16 ms) position, with a mean difference of 3.67 ms (95% CI [3.52, 3.81]). However, PT relaxation time was shorter in the flexed position (5.65 ± 0.10 ms) than in the normal and extended positions, with a mean difference of 4.4 ms (95% CI [4.24, 4.55]) and 0.73 ms (95% CI [0.65, 0.80]), respectively (F [1.3,32] = 4,242.062; P < 0.001). Similarly, in the muscle relaxation group, PT relaxation time was longer in the normal position (12.10 ± 0.71 ms) than in the extended position (8.36 ± 0.62 ms), with a mean difference of 3.74 ms (95% CI [3.17, 4.31]). Nevertheless, PT relaxation time was shorter in the flexed position (7.30 ± 0.49 ms) than in the normal and extended positions, with a mean difference of 4.8 ms (95% CI [4.46, 5.12]) and 1.06 ms (95% CI [0.69, 1.41]), respectively (F [1.2,31] = 445.094; all P < 0.001).

### Creep

**Fig 7:**
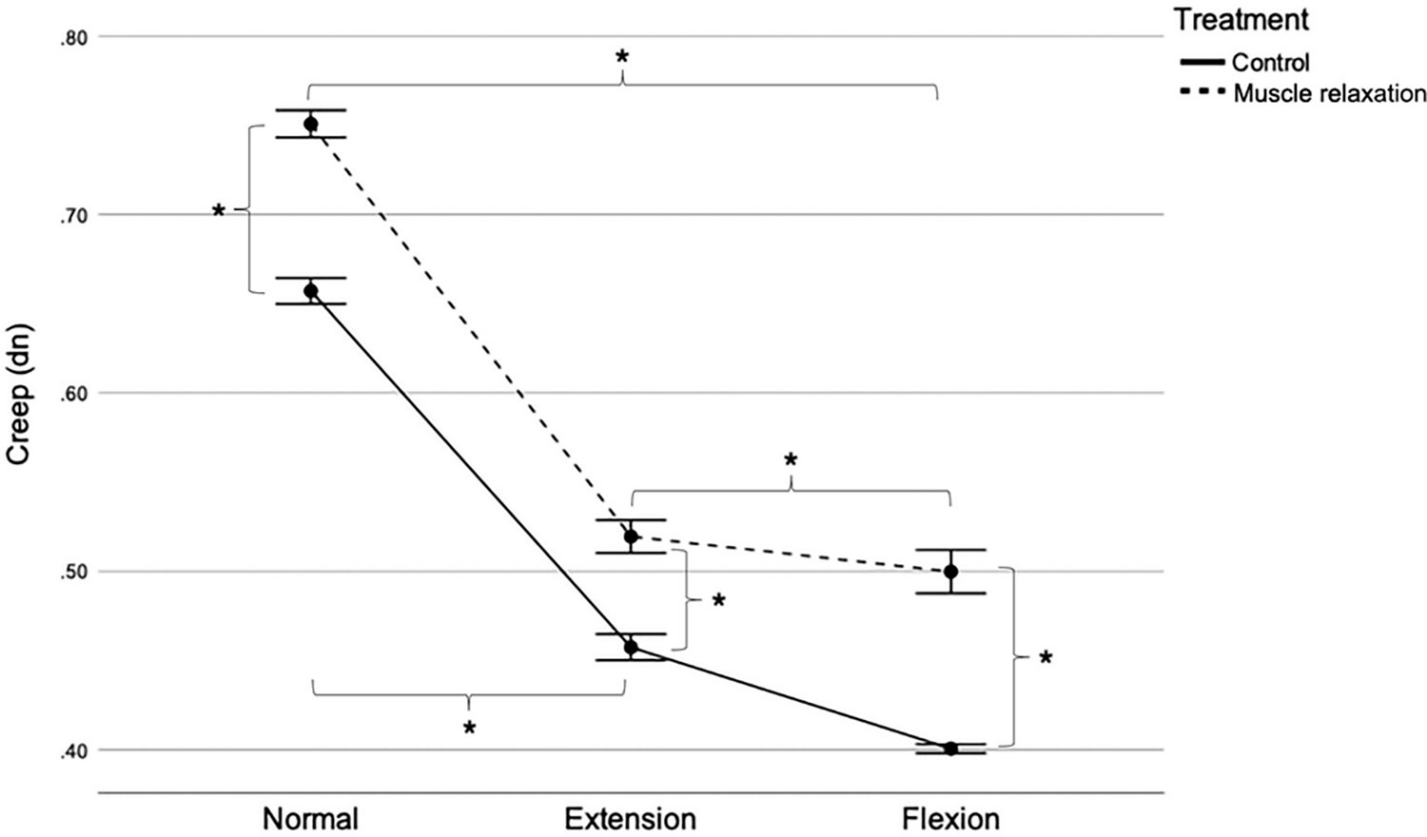
Patellar tendon creep in the control and muscle relaxation groups. Mean with SEM. *P < 0.05. dn, Deborah number; SEM, standard error of the mean.

Fig 7 shows the PT creep values between the control and muscle relaxation groups at various stifle angle positions. Two-way RM ANOVA revealed significant interactions between treatment and position (F [2, 48] = 26.941; P < 0.001). The effects of the different treatments on creep depended on the stifle angle position. In the normal position, PT creep was significantly lower in the control group (0.65 ± 0.01 dn) than in the muscle relaxation group (0.75 ± 0.01 dn) (F [1,24] = 259.251; P < 0.001), with a mean difference of 0.1 dn (95% CI [0.08, 0.10]). Similarly, in the extended position, PT creep was significantly lower in the control group (0.45 ± 0.01 dn) than in the muscle relaxation group (0.51 ± 0.02 dn) (F [1,24] = 137.948; P < 0.001), with a mean difference of 0.06 dn (95% CI [0.05, 0.07]). Furthermore, in the flexed position, PT creep was also significantly lower in the control group (0.40 ± 0.01 dn) than in the muscle relaxation group (0.49 ± 0.03 dn) (F [1,24] = 214.179; P < 0.001), with a mean difference of 0.09 dn (95% CI [0.08, 0.11]).

The interaction also indicated that the effect of stifle angle position on PT creep varied depending on the treatment. In the control group, PT creep was higher in the normal position (0.65 ± 0.01 dn) than in the extended position (0.45 ± 0.01 dn), with a mean difference of 0.2 (95% CI [0.18, 0.21]). However, PT creep was lower in the flexed position (0.40 ± 0.01 dn) than in the normal and extended positions, with a mean difference of 0.25 dn (95% CI [0.24, 0.26]) and 0.05 dn (95% CI [0.04, 0.06]), respectively (F [1.2,30] = 2,260.501; all P < 0.001). Similarly, in the muscle relaxation group, PT creep was higher in the normal position (0.75 ± 0.01 dn) than in the flexed extension (0.51 ± 0.02 dn), with a mean difference of 0.24 dn (95% CI [0.22, 0.24]). Nevertheless, PT creep was lower in the flexed position (0.49 ± 0.03 dn) than in the normal and extended positions, with a mean difference of 0.26 dn (95% CI [0.23, 0.26]) and 0.02 (95% CI [0.01, 0.03]), respectively (F [2, 48] = 1,820.527; all P < 0.001).

### Correlations between body weight and PTL

Table 3 demonstrates the correlation between body weight and PTL in the normal, extended, and flexed positions.

**Table 3:** Correlation between body weight and patellar tendon length.

		1	2	3	4
1.	Body weight				
2.	Normal	0.84			
3.	Extension	0.83	0.91*		
4.	Flexion	0.81	0.90*	0.99**	

* p < 0.05

** p < 0.01

## Discussion

The current study is the first to quantify PT’s biomechanics and viscoelastic properties in living dogs using Myoton devices. The primary findings showed that the position of the stifle angle and the NMBA affected dog PT’s biomechanical and viscoelastic qualities. In addition, the relationship between body weight and PTL (Table 3) using Pearson’s r statistic was not statistically significant. The author believes the lack of statistical significance might have been caused by the limited sample size (N = 5). The research involved six canines of comparable ages, with a particular emphasis on female subjects to reduce potential sources of variation. The author believes that gender and age could influence outcomes, drawing parallels with human medicine. Despite the exclusive focus on female dogs, the study’s pioneering methodology and baseline data can form a strong foundation for future research.

The administration of muscle relaxants resulted in a notable reduction in tone, stiffness, and decrement. The authors postulate that muscle relaxant was unlikely to impact tendons directly due to their primer influence on muscle relaxation. Around the stifle joint, the quadriceps femoris and the posterior muscle compartment (plantaris, articulus genu, semitendinosus, semimembranosus, and popliteus) are responsible for joint stabilization and movement. Furthermore, by preventing muscle contraction, the PT experiences less stress and strain, decreasing tone, stiffness, and decrement. The explanation aligns with the reference by [18–23], which states that the loading conditions or forces occurring on a tendon are controlled by the surrounding muscles, which attach to the tendon and contract to generate force. Multiple studies have also provided evidence of the efficacy of muscle relaxants in canines undergoing orthopedic surgery [24–27]. This highlights its essential role in optimizing muscle relaxation during medical procedures, indirectly affecting tendon function and overall joint stability. The relaxation time and creep were significantly lower in the control group than in the muscle relaxation group. Tissues with higher stiffness might rapidly influence the condition to return to their original shape. A faster relaxation time indicates that the tendon returns to its initial length more quickly after being stretched. The time-dependent behavior of materials under applied stress or strain, widely observed in biological tissues, is called viscoelasticity. The association between tissue stiffness and viscoelastic qualities has been reported in several studies [9, 28–30]. Tissues with higher stiffness tend to have slower relaxation rates and creep under constant stress or load. The observed disparities in relaxation times and creep values between the groups indicate the complex viscoelastic nature inherent to these biological components, underscoring the broader implications of tissue mechanics within physiological contexts.

The evaluation of PT tone in various positions revealed notable differences within the control and muscle relaxation groups. Our studies indicated that PT tone was the lowest at normal position and the shape was not straight or elongated (Fig 2). The absence of elongation suggests a state of rest, corroborated by the lower tension observed. This aligns with the findings of another study [14], where dogs displayed minimal PT tension while standing, emphasizing how their natural posture fosters relaxation in their physiological state. The highest value was observed in the flexion for both groups. This observation can be attributed to the mechanics of knee flexion, wherein the patella is displaced away from the femur, facilitating the lengthening and stretching of the tendon. Notably, PT elongation is shown in flexion and extension positions (Table 1). However, it was exclusively during knee flexion that the PT tone displayed its peak value. The phenomenon might be attributed to the heightened stress experienced by the PT in the flexed position compared to the other positions. In line with this notion, previous research [31–33] using elastography reported that PT exhibits more significant shear strain and stiffness during flexion, reflecting higher passive tension generated by the surrounding musculoskeletal structures. Indeed, a study by [8] using a Myoton device showed that PT tone at 90° (flexion) was the highest compared to that at 0°, 30°, and 60° degrees.

The stiffness results in the flexion position were the highest in both the control and the muscle relaxation groups across all positions. Similar to the tone parameter, the PT experienced the highest stiffness in the flexed position due to the tendon being stretched (Fig 2). These results are in accordance with [34], which states that the gastrocnemius muscles become stiff due to a greater dorsiflexion angle during static stretching. Another study [10] showed that AT measurements taken with the foot in 0° of dorsiflexion significantly increased stiffness compared to those obtained when the subject was standing or in a relaxed position (hanging freely over the edge of the examination bed). Given the more linear explanation, the measurements in this research were taken at the PT’s midpoint, which was subjected to the highest stress. This argument is consistent with previous observations, which reported that tendon stress would be higher at the middle of the PT (roughly 50% of the length), approximately 20–25 mm from the patella’s apex [35]. Other investigations also found that the structure of the PT and subcutaneous fat, which generate PT strain when the knee is flexed, is strongly associated with the higher stiffness values of the PT as the knee angle increases [8]. Research using elastography has confirmed that the relative stiffness for both the PT and quadriceps muscles increases as the knee approaches its maximum flexion angle [4].

Regardless of the position, the most significant decrement values were found in the flexion position for both groups. Decrement parameters from the Myoton device were used to inversely describe elasticity, suggesting that as the value of decrement increases, the elasticity of the tissue decreases. As shown in Table 2, a similar pattern was seen in the current study. The association can be highlighted by the fact that when the tissue becomes stiff, its ability to deform and return to its normal shape decreases. Stiffness and elasticity are typically inversely related in biological tissues, including tendons and cartilage. These tissues comprise a dense network of fibers or proteoglycans that resist deformation [36]. When subjected to strain or stretch, tendon fibers become denser and less flexible. These alterations impair the tendons’ ability to deform when a force is applied and their capacity to stretch and recover, leading to a loss of elasticity [19]. In other words, increased stiffness in biological tissue is often accompanied by a decrease in elasticity as the tissue becomes less able to deform under load and dissipate energy. The findings are congruent with [37] on the masseter muscle and [38] on the rectus femoris and biceps brachii. Their results revealed that the elasticity decreases as the tone and stiffness of muscles increase.

The current study shows that flexion in both groups produced the shortest relaxation time. Conversely, relaxation time was consistently the longest in the normal position for both groups. The essential assumption is that the PT is an example of a biological tissue that exhibits both elasticity and viscosity. When force is applied to a tendon, it deforms and stores energy elastically. As strain is prolonged, the tendon becomes viscous, losing energy during deformation and relaxation. This ability would give the PT a shorter relaxation time than other tissues, allowing them to quickly restore their original shape and mechanical properties when subjected to repeated stress. This finding is consistent with previous research demonstrating the relationship between tone/stiffness and relaxation time. The observations revealed that the relaxation time was considerably shorter when the tissue/muscle had a higher tone and stiffness [9, 39–41].

Similar to the relaxation time, creep was the lowest during flexion in both groups. In contrast, creep was the highest in the normal position for both groups. Creep represents the tendency of a material to continue to deform over time when subjected to constant load or stress. The author’s perspective was that there was an inverse correlation between stiffness and creep due to the viscoelastic properties of the PT. Earlier research [42] identified a correlation between water and creep on ligament viscoelasticity. This study suggests that decreasing water content in the ligaments would result in a reduced viscoelastic response and creep. Further investigations have indicated that removing water molecules around collagen fibrils alters its microarchitecture and increases tissue stiffness [43]. The PT experienced the highest stiffness during flexion compared to other positions, i.e., increased stiffness will decrease the amount of water molecules around the collagen fibers. This assumption is aligned with a previous investigation [44] that stated that a stiffer PT would restrict water from entering or remaining within the fibers by limiting the movement of water molecules. This phenomenon is evidenced by squeezing the water molecules out or reducing the space between collagen fibers by a compression mechanism. Additionally, several prior studies have suggested an inverse correlation between creep and tone/stiffness in tissues. As tone and stiffness increase, creep decreases [42, 45, 46].

## Conclusions

The study demonstrates that muscle relaxants and the stifle joint angle may alter the biomechanical and viscoelastic properties of the PT. Analysis of the dog’s PT tone, stiffness, decrement, relaxation time, and creep reveal distinct responses to joint angles, particularly heightened tone and stiffness with reduced elasticity during flexion. Thus, it enhances our comprehension of tissue biomechanics by emphasizing interconnected mechanical parameters, particularly in veterinary medicine.

## Limitations

This study contains some limitations that should be highlighted. The exclusive focus on female dogs may restrict the generalizability of findings. Furthermore, it was practically difficult to manage the dog without anesthesia while measuring all parameters under various stifle angle positions. Additionally, various dog breeds should be examined to ensure the results possess a broader scope.

## Supporting information

S1 Table Measurement of the biomechanical and viscoelastic characteristics of the patellar tendon. (XLSX)
